# Risk factors for long-term arm morbidities following breast cancer treatments: A systematic review

**DOI:** 10.18632/oncotarget.28539

**Published:** 2023-12-01

**Authors:** Ifat Klein, Michael Friger, Merav Ben David, Danit Shahar

**Affiliations:** ^1^Department of Physical Therapy, Assuta Medical Center, Ramat Hahayal, Tel Aviv 6971028, Israel; ^2^Department of Epidemiology, Biostatistics and Community Health Sciences, School of Public Health, Faculty of Health Sciences, Ben-Gurion University of the Negev, Beer Sheva 8410501, Israel; ^3^Faculty of Health Sciences, Ben-Gurion University of the Negev, Beer Sheva 8410501, Israel

**Keywords:** risk factors, arm morbidity, physical rehabilitation, breast cancer

## Abstract

Purpose: To examine the risk factors for arm morbidity following breast cancer treatments, taking a broad view of all types of physical morbidity, including prolonged pain, lymphedema, decreased range of motion, and functional limitations.

Methods: A systematic literature review was performed according to Preferred Reporting Items for Systematic Reviews and Meta-Analyses (PRISMA) Guidelines. Studies exploring the risk factors for prolonged arm morbidity following breast cancer surgery and treatments were included. The studies were assessed independently according to pre-eligibility criteria, following data extraction and methodological quality assessment.

Results: 1,242 articles were identified. After removing duplicates, the full texts of 1,153 articles were examined. Sixty-nine of these articles met the criteria and were included in the review. These 69 articles identified 29 risk factors for arm morbidity following treatments for breast cancer. The risk of bias was evaluated using NIH study quality assessment tools. The studies reviewed were published between 2001 and 2021 and included a total of 22,886 patients who were followed up for between three months and 10 years.

Conclusions: The main risk factors for long-term morbidity are removal of lymph nodes from the axilla, body mass index >30, having undergone a mastectomy, the stage of the disease, radiation therapy, chemotherapy, infection and trauma to the affected arm after surgery. An understanding of the risk factors for prolonged arm morbidity after surgery can help doctors and therapists in making personalized decisions about the need and timing of rehabilitation treatments.

## INTRODUCTION

Breast surgery and the various oncological treatments for breast cancer (BC), treat the disease and save lives, but may nonetheless have adverse effects that may last years after diagnosis [1–3].

The physical rehabilitation after BC treatment requires a broad view of the set of problems that patients suffer from [4]. Prolonged pain defined as pain that lasts up to three months after surgery, which is felt in the breast, armpit or arm are the most common problems and occur in up to 50% of patients [5, 6]. Functional limitations occur in up to 50% of patients and affect their quality of life [2]. Pain and functional limitations are the main causes of women’s difficulty in returning to the jobs they held pre-diagnosis, and apply to up to 48% of patients [7]. Secondary lymphedema (chronic edema) due to lymph node dissection and radiation treatment may develop over months and gradually become a chronic condition with a prevalence of up to 17% [8]. Cognitive impairment and fatigue are common in up to 25% and 30% of patients, respectively [9]. The prevalence of sleep difficulties ranges between 20% and 70% [10]. Two additional symptoms are chemotherapy-induced peripheral neuropathy (30%), and cardiotoxicity 6–17% [11]. All the above symptoms affect survivors’ quality of life [12] and require long emotional and physical rehabilitation [13].

Identifying patients at risk of arm morbidity and providing rehabilitation programs can help survivors throughout their journey to recovery [14]. Therefore, the importance of understanding the risk factors and their effect on morbidity, including health behaviors, the type of oncological treatments including type of surgery, and even the patient’s emotional state, increases [15].

Several tools have been developed to assess the risk of morbidity, such as the RATE-L, which assesses the risk of lymphedema using known risk factors, including body weight, age, number of lymph nodes removed, axillary radiation therapy and chemotherapy. [16].

Other risk factors are known to affect the risk of developing a limitation in the range of motion (ROM), pain or causes a decrease in function [17, 18]. A thorough understanding of the impact of the various risk factors resulting from BC treatments and their effect on arm symptoms can empower doctors and therapists to optimize treatment protocols and identify patients who need symptom monitoring or preventive [19, 20].

We hypothesize that understanding the risk factors for arm morbidity after BC treatments, including personal characteristics, various aspects related to surgery and oncological treatments, could lead to the design of tools and programs, intend to identify patients at risk at an earlier stage and thereby provide better care [21, 22].

Therefore, the aim of this systematic review is to identify risk factors for the four most common physical symptoms by patients, namely, persistent pain, lymphedema, decreased ROM, and functional disability.

## RESULTS

**Figure 1:**
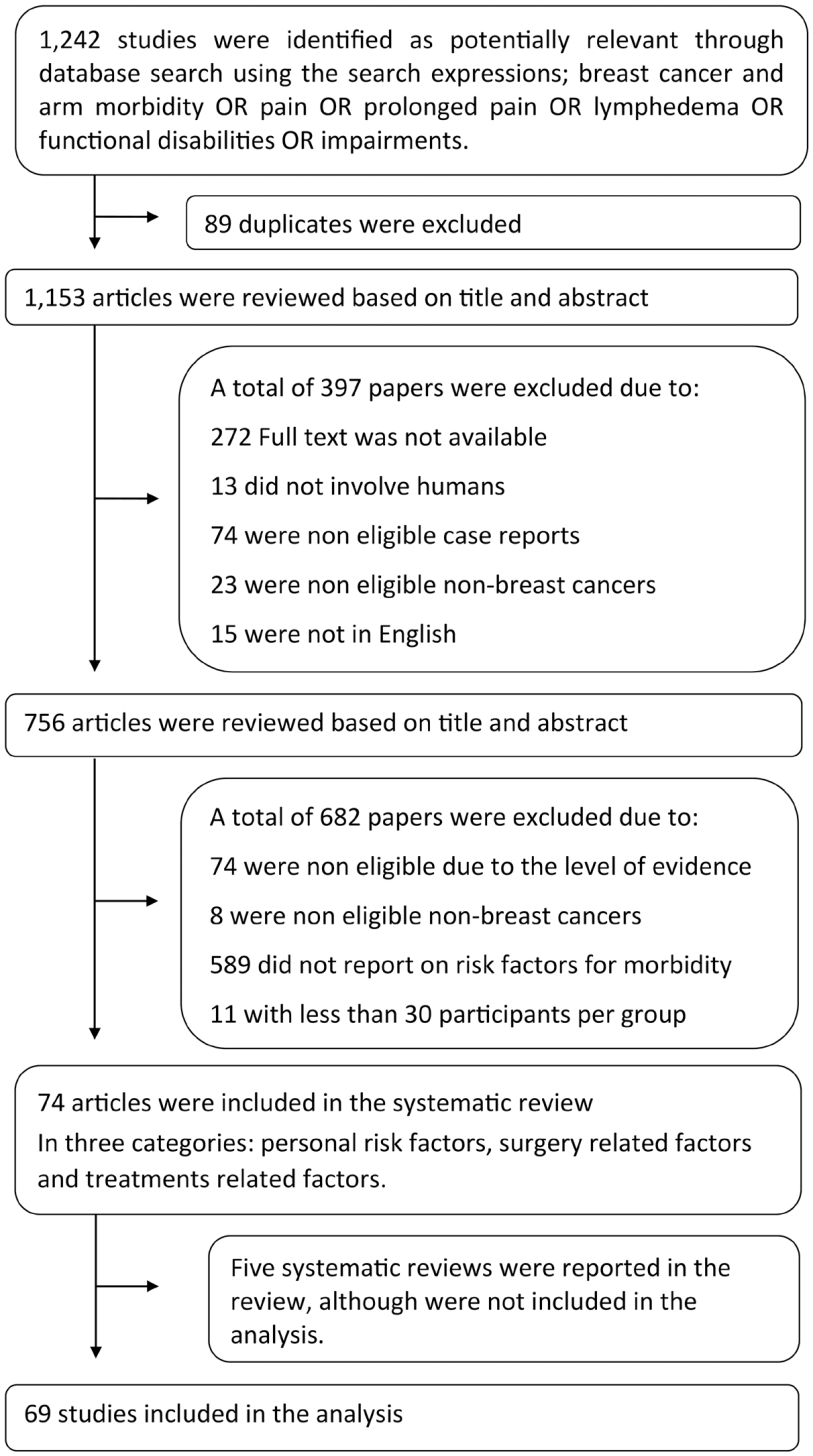
Flow chart of the study selection. Search process and selection of articles according to the preferred reporting items for systematic reviews and meta-analyses (PRISMA) Guidelines [102].

**Figure 2:**
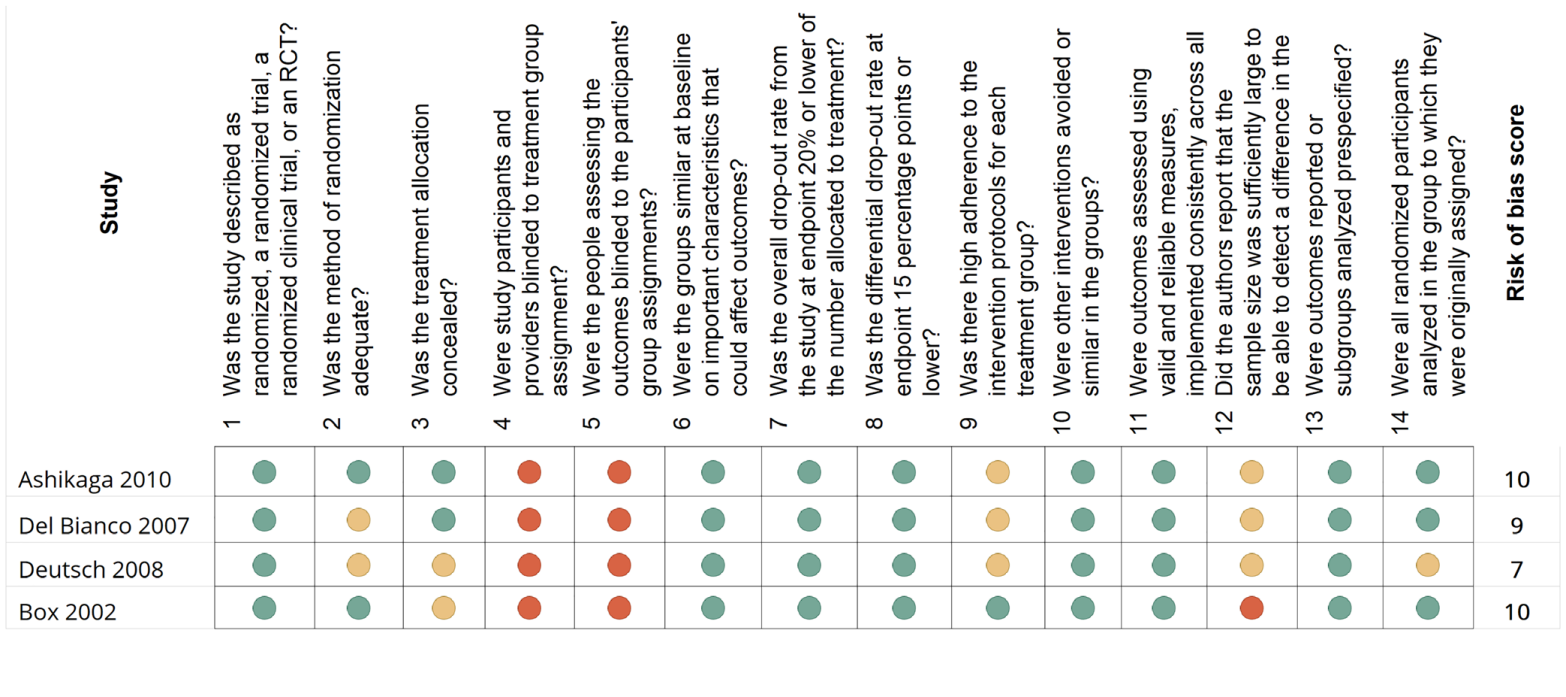
A summary and risk-of-bias assessment according to the National Institutes of Health quality assessment tool for randomized control studies (https://www.nih.gov/). This figure describes the risk of bias in each of the 65 observational studies, cohort studies, and cross-sectional studies included in the review. The National Institute of Health (NIH) risk-assessment tool includes 14 questions evaluating bias risk. This diagram represents the answer options; green indicates a positive answer; red indicates a negative answer; yellow indicates questions that are cannot be determined or not.

**Figure 3:**
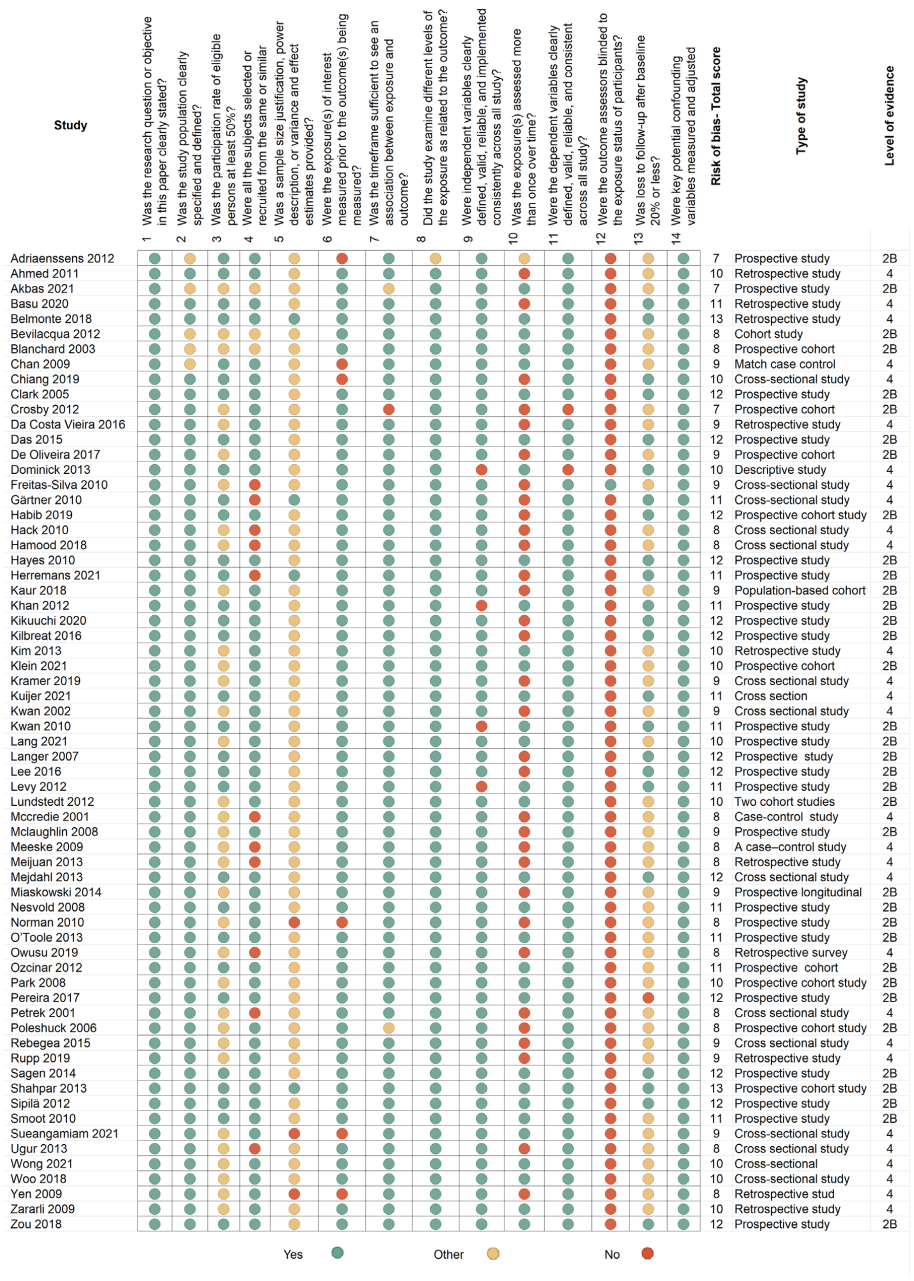
A summary and risk-of-bias using the quality assessment tool for observational cohort and cross-sectional studies (https://www.nih.gov/). This figure describes the risk of bias in each of the 65 observational studies, cohort studies, and cross-sectional studies included in the review. The National Institute of Health (NIH) risk-assessment tool includes 14 questions evaluating bias risk. This diagram represents the answer options; green indicates a positive answer; red indicates a negative answer; yellow indicates questions that are cannot be determined or not applicable.

After conducting electronic searches based on abstracts and titles, we identified 1242 articles. After removing duplicates, 1,153 articles were examined for full text. Sixty-nine of these articles met the criteria and were included in the review. Flow chart of the study is shown in Figure 1. The literature reviews included five systematic reviews, four RCTs and 65 studies with evidence quality levels of 1A to 2B. The classification of evidence is presented in Table 1 and the risk of bias assessments are described in Figure 2 and Figure 3 according to study type.

**Table 1:** Classification of evidence system based on the Oxford Centre for Evidence-Based Medicine (OCEBM) Levels of Evidence

1A	Systematic review (with homogeneity) of RCTs
1B	Individual RCT (with narrow confidence intervals)
2A	Systematic review (with homogeneity) of cohort studies
1C	All or none study
2B	Individual cohort study including low quality RCT
2C	Outcomes research; ecological studies
3A	Systematic review (with homogeneity) of case-control studies
3B	Individual case-control study
4	Case series (and poor-quality cohort and case-control study)
5	Expert opinion without explicit critical appraisal or based on physiology bench research or “first principles”

**Figure 4:**
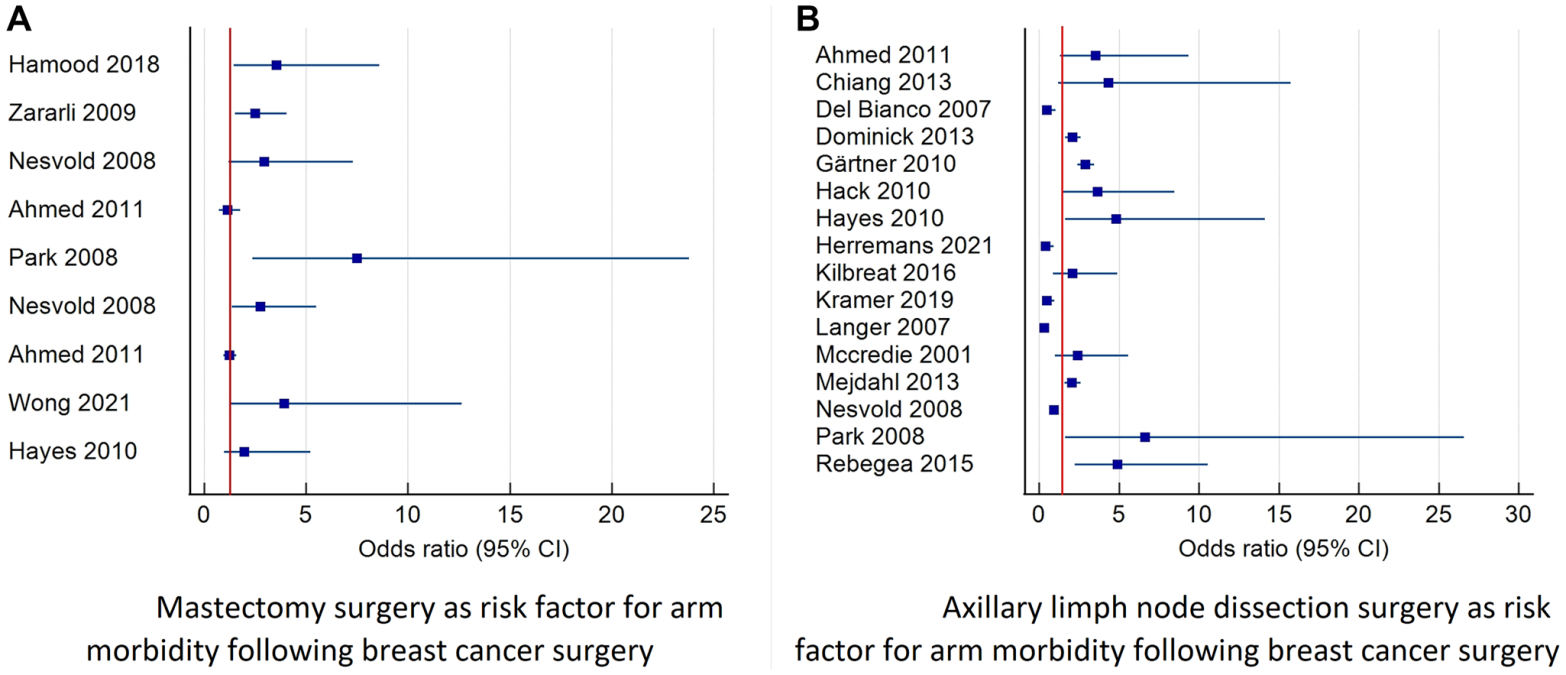
Forest plot diagram of odds ratio (95% CI) for surgery related factors as risk factors for prolonged arm morbidity. This figure describes studies that reported the odds ratio and 95% confidence interval for mastectomy and axillary surgery as risk factors for combined morbidity of the arm including: pain, lymphedema, decreased function and decreased range of motion. (**A**) Describes the risk of morbidity after mastectomy surgery. (**B**) Describes the risk of morbidity after axillary lymph node dissection. Abbreviation: CI: confidence interval.

**Figure 5:**
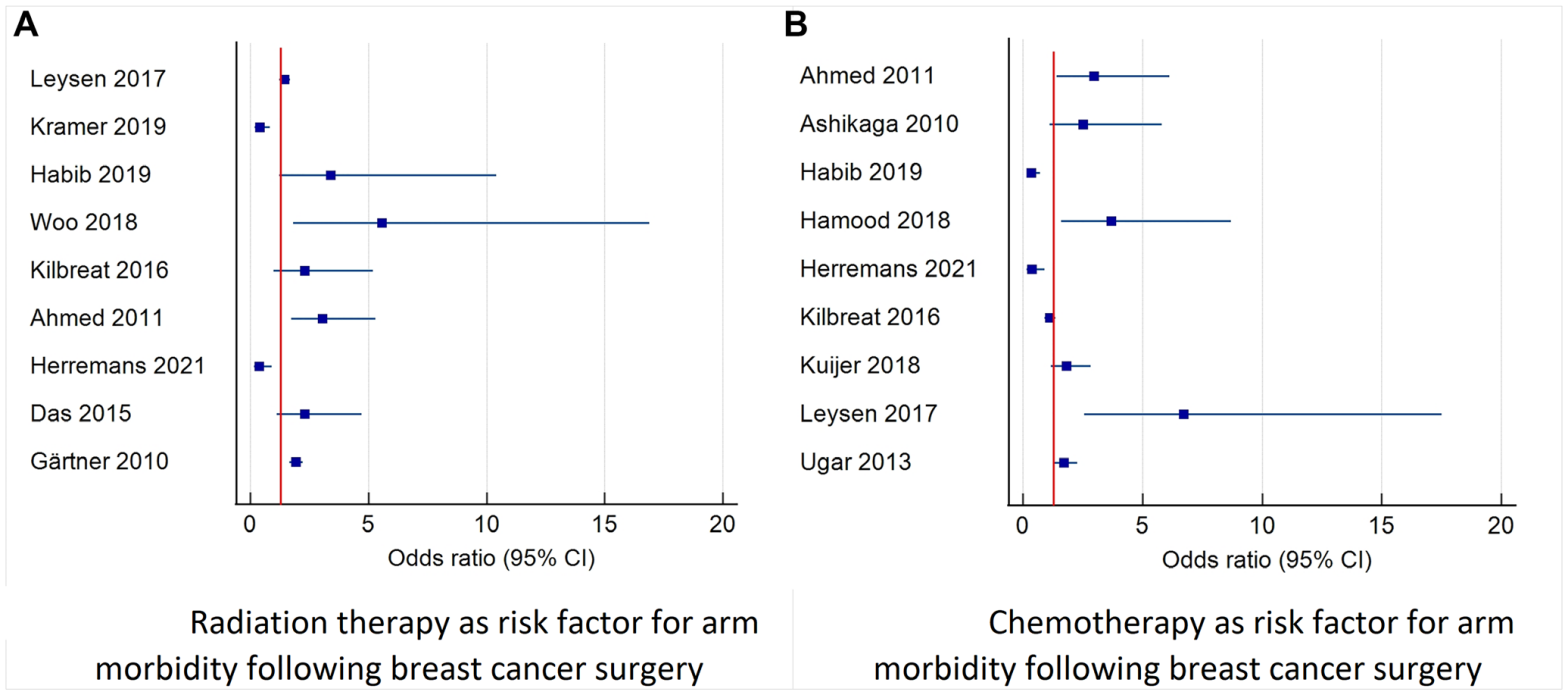
Forest plot diagram of odds ratio (95% CI) for risk factors for arm morbidity related to oncological treatment. This figure describes studies that reported the odds ratio and 95% confidence interval for radiotherapy and chemotherapy as risk factors for combined morbidity of the arm including: pain, lymphedema, decreased function and decreased range of motion. (**A**) Describes the risk of radiation therapy morbidity. (**B**) Describes the risk of morbidity after chemotherapy (adjuvant and neoadjuvant). Abbreviation: CI: confidence interval.

The study included articles published between 2001 and 2021, with a total of 22,886 patients and a follow-up period of between three months and ten years. The results were divided into the following three categories: (1) personal risk factors; (2) risk factors related to surgery; and (3) factors related to oncological treatments (See Tables 2–8 and Figures 4, 5).

**Table 2:** Young age as a risk factor for arm morbidity following breast cancer treatments

Disability	Author	*N*	Follow-up	Measure	Comparison/Comments	*P*-value	OR/RR/HR	CI (95%)	Type of evidence	Evidence level
Persistent pain	Wang 2016 [25]	11,030	Median follow-up 12 months	VAS)	OR for every 10 years		OR = 1.36	1.24–1.48	Systematic review and meta-analysis	2A
	Miaskowski 2014 [23]	398	6 months	ASQ and Postsurgical Pain Questionnaire	No pain Mild pain Moderate pain	<0.001			Prospective longitudinal study	2B
	Poleshuck 2006 [26]	95	3 months	NPRS		0.004	OR = 0.95	0.91–0.99	Prospective study	2B
	Zararli 2009 [32]	299		AVS	Age <50 vs. >50	0.001	RR = 2.493	1.532–4.058	Prospective study	2B
	Lee 2016 [30]	375		Brief Pain Inventory and NPRS	Age <50 vs. >50	0.010	OR = 2.44	1.24–4.79	Prospective study	2B
	Lundstedt 2012 [31]	1,027	BC 3 to 20 years earlier	Based on Lundstedt questionnaire	Breast pain vs. no breast pain following radiation		RR = 0.96	0.94–0.98	Two cohort studies data collection	2B
	Mejdahl 2013 [27]	2,411	6 years	NPRS	With or without chronic pain	<0.001	OR = 1.78	1.25–2.5	Repeated cross-sectional study	4
	Kaur 2018 [28]	215	3 months	VAS and Neuropathic Pain Symptom Inventory	<3/>3 Visual analog scale	0.03			Descriptive study	4
	Meijuan 2013 [29]	225	-	McGill Pain Questionnaire and SF36		<0.05			Retrospective study	4
	Hamood 2018 [24]	410	7.4 years	NPRS	With or without chronic pain	0.002	OR per one year = 0.96	0.94–0.99	A cross-sectional study	4
Functional disabilities	Zararli 2009 [32]	299		Physical examination	Age <50 vs. >50	0.048	RR = 2.493	1.53–4.05	Prospective study	2B
	Khan 2012 [33]	85		Functional Independence Measure	<57 vs. >57	0.007			Prospective study	2B
	De Oliveira 2017 [34]	101	12 months after surgery	The DASH questionnaire	<56 vs. 57<	0.035		1.3–7.8	Retrospective cross-sectional study	4
Lymphedema	Meeske 2009 [35]	494	50 months after diagnosis	Self-report	Age 35–44, 45–54, 55–64		OR per year of age = 0.96	0.93–0.99	A case-control study	4
	Gärtner 2010 [36]	3,253		Self-report	Age 18–39, 40–49, 50–59, 60–69	<0.001	OR = 2.60	1.72–3.94	cross-sectional study	4
Axillar web syndrome or cording	O’Toole 2013 [38]	308	8 months	Perometer and the LEFT-BC questionnaire Cording by patient self-report		0.005	HR = 0.96	0.94–0.98	Prospective study	2B

Abbreviations: *N*: number; CI: confidence interval; OR: odds ratio; HR: hazard ratio; RR: relative risk; BMI: body mass index; ALND: axillary lymph node dissection; sentinel lymph node biopsy; VAS: visual analog scale; ROM: range of motion, ASQ: shoulder symptoms questionnaire; NPRS numeric pain rating scale; RCT: randomize control trail.

**Table 3:** Age over 50 as a risk factor for arm morbidity following breast cancer treatments

Disability	Author	*N*	Follow-up	Measure	Comparison/Comments	*P*-value	OR/RR/HR	CI (95%)	Type of evidence	Evidence level
Functional disabilities	Hayes 2010 [39]	287	18-months	*QuickDASH*	<50, 50>	<0.05	OR = 1.94	1.01–3.75	Prospective study	2B
Lymphedema	Ashikaga 2010 [37]	3,963	36-months	Arm volume	ALND vs. SLNB and age <49, 50<	0.006	OR = 1.41		RCT	1B
	Bevilacqua 2012 [42]	1,051	5 years	Arm circumference measurement	Age <50/50>	0.0485			Prospective cohort study	2B
	Herremans 2021 [43]	132	5 years	Circumference measurement and bioimpedance spectroscopy	LYMPHA technique and ALND	0.037	OR = 0.37	0.14–0.94	Retrospective cross-sectional study	4
	Chan 2009 [41]	202 after ALND		Arm circumference measurement	Level of lymphedema increases with age	0.011	OR = 1.05	1.01–1.10	Matched cases control study	3B
Persistent pain	Sipilä 2012 [44]	489	6 months	NPRS	Age <39, 40–69, 70<	<0.01	OR = 0.35	0.16–0.76	Prospective study	2B
	Mejdahl 2013 [27]	2,411		Developed questionnaire	For age 60–69	<0.001	OR = 1.12	0.90–1.41	Repeated cross-sectional study	4
Decreased range of motion	Woo 2018 [45]	430		Having restricted ROM ≥ 30° in comparison to the unaffected side	Different modalities of breast reconstructions	0.041	OR = 1.038	1.00–1.07	Prospective study	2B
	Levy 2012 [40]	115	12 months	Harvard Alumni Health Study Physical Activity Questionnaire and The *QuickDASH* Questionnaire	Severity of limitation	0.001			Prospective study	2B

Abbreviations: *N*: number; CI: confidence interval; OR: odds ratio; HR: hazard ratio; RR: relative risk; BMI: body mass index; ALND: axillary lymph node dissection; sentinel lymph node biopsy; VAS: visual analog scale; ROM: range of motion, ASQ: shoulder symptoms questionnaire; NPRS numeric pain rating scale; RCT: randomize control trail.

**Table 4:** Body mass index (BMI) as risk factor for arm morbidity following breast cancer treatments

Disability	Author	*N*	Follow-up	Measure	Comparison/Comments	*P*-value	OR/RR/HR	CI (95%)	Type of evidence	Evidence level
Lymphedema	Deutsch 2008 [51]	1,457		Arm circumferences		0.001			RCT	1B
	Box 2002 [57]	65	24 months	Arm circumferences, arm volume and multi-frequency bioimpedance		0.01	OR = 1.21	1.04–1.41	RCT	1B
	Nesvold 2008 [48]	263	47 months	Volume calculation		<0.01	OR = 1.11	1.04–1.19	Prospective study	2B
	Kwan 2010 [49]	997	20.9 months	Examination by a specialist	BMI 25 (healthy weight) 25–29 (overweight) >30 (obese)		HR = 1.43	0.88–2.31	Prospective study	2B
	Zou 2018 [47]	387	24 months	Circumference and Norman questionnaire	BMI <24/24<	0.03	HR = 1.1	1.0–1.1	Prospective study	2B
	Shahpar 2013 [50]	410	3 years	Arm circumferences	Increase of 1 kg/m2 in Body mass index	<0.001	OR = 1.09	1.05–1.15	Prospective cohort study	2B
	Das 2015 [55]	199	2–15-year	Self-reported	BMI> = 25 treated with tamoxifen	0.05	OR = 2.62	0.99–6.93	Prospective cohort	2B
	Mclaughlin 2008 [52]	936	5 years	Circumference		<0.001			Prospective study	2B
	Kilbreath 2016 [53]	450	18-months	Bio-impedance spectroscopy	<5/>5 lymph nodes removed	0.08	OR = 1.9	0.9–3.9	Prospective cohort	2B
	Kuijer 2021 [17]	1,037	1-year	Self-reported	BMI 25–30	0.03	OR = 1.7		Prospective cohort study	2B
	Bevilacqua 2012 [42]	1,051	5 years	Arm circumference measurement	BMI <25/>25	0.001			Prospective cohort	2B
	Chan 2009 [41]		5 years	Arm circumference measurement		0.007	OR = 1.11	1.03–1.21	Matched case-control study	3B
	Meeske 2009 [35]	494	50 months after diagnosis	Self-report of lymphedema			OR for, BMI>30 = 2.48	1.05–5.84	A case-control study	4
	Petrek 2001 [54]	923	20 years	Circumferential arm measurements		0.08			Cross-sectional	4
	Mccredie 2001 [56]	809		Self-reported	For BMI>30	0.02	OR = 1.7	1.1–2.6	Population-based study	4
	Smoot 2010 [104]	144		The DASH questionnaires and circumference measurement		0.041		−3.68–−0.08	Cross-sectional study	4
Lymphedema BMI>25	Nesvold 2008 [48]	263	47 months	British Columbia Cancer Agency in Canada		<0.01	OR = 1.12	1.04–1.19	Prospective study	2B
	Dominick 2013 [61]	2,431	6 years	Norman and colleagues’ validated telephone lymphedema questionnaire	For BMI>30	<0.01	OR = 2.08	1.66–2.60	Prospective cohort study	2B
	Ugur 2013 [62]	455	53 months	Tape measure	BMI <25/>25	<0.001	OR = 3.94	1.97–7.87	Prospective study	2B
	Park 2008 [63]	450	24 months	Tape measure	BMI <25/>25	0.023	OR = 2.01	1.10–3.68	Prospective study	2B
	Clark 2005 [64]	251	3 years	Tape measure	BMI <25/>25		RR = 2.02	1.11–3.68	Prospective observational study	2B
	Crosby 2012 [58]	1,117		From medical record	Immediate breast reconstruction and BMI <25/>25	0.008	OR = 2.78	1.30–5.94	Retrospective study	4
Functional disabilities	Nesvold 2008 [48]	263	47 months	British Columbia Cancer Agency in Canada		0.001	OR = 1.12	1.05–1.2	Prospective study	2B
	Hack 2010 [59]	316	12 months	The DASH Questionnaire		0.026			Cross-sectional study	4
Decreased range of motion	Kuijer 2021 [17]	1,037	1-year	CARES-SF	For BMI 25–30	0.05	OR = 1.5		Multicenter prospective cohort study	2B
	Levy 2012 [40]	115	12 months	Harvard Alumni Health Study Physical Activity Questionnaire	BMI <25/>25	<0.05			Prospective study	2B
	Basu 2020 [60]	342	37 months	Questionnaire was inspired by the Australian BC Family Study	BMI <25/>25	0.04	HR = 1.42	1.6–2.4	Retrospective study	4
Persistent pain	Laurence 2017 [65]					0.008	OR = 1.34	1.08–1.67	Meta analysis	1A
	Sipilä 2012 [44]	489	6 months	NPRS	BMI <25, 26–30, >30	<0.01	OR = 0.58	0.34–0.98	Prospective study	2B

Abbreviations: *N*: number; CI: confidence interval; OR: odds ratio; HR: hazard ratio; RR: relative risk; BMI: body mass index; NPRS: numeric pain rating scale; RCT: randomize control trail.

**Table 5:** Mastectomy as a risk factor for arm morbidity following breast cancer treatments

Disability	Author	*N*	Follow-up	Measure	Comparison/Comments	*P*-value	OR/RR/HR	CI (95%)	Type of evidence	Evidence level
Persistent pain	Nesvold 2008 [48]	263	47 months	British Columbia Cancer Agency in Canada		0.001			Prospective study	2B
	Pereira 2017 [69]	506	12 months	Brief Pain Inventory, Pain Disability Index	Mastectomy with ALND		RR = 2.52	1.25–5.11	Cohort study	2B
	Hamood 2018 [24]	410	7.4 years	NPRS	With or without chronic pain	0.005	OR = 3.54	1.46–8.59	A cross-sectional study of a random sample	4
Paresthesia	Zararli 2009 [32]	299		Self-report		0.048	RR = 2.493	1.53–4.05	Prospective	2B
Lymphedema	Hack 2010 [59]	316	24 months	Circumference and the Norman questionnaire			HR = 2.1	1.3–3.4	Prospective study	2B
	Nesvold 2008 [48]	263	47 months	Volume calculation		0.02	OR = 2.94	1.2–7.3	Prospective study	2B
	Clark 2005 [64]	251	3 years	Tape measure			RR = 2.04	1.18–3.54	Prospective observational study	2B
	Park 2008 [63]	450	24 months	Tape measure		0.035	OR = 7.48	2.38–23.85	Prospective study	2B
	Ahmed 2011 [71]	1,287		Self-report			OR = 1.13	0.72–1.77	Cross-sectional study	4
Decreased range of motion	Nesvold 2008 [48]	263	47 months	British Columbia Cancer Agency in Canada		0.04	OR = 2.75	1.37–5.5	Prospective study	2B
	Kuijer 2021 [17]	1,037	1 year	CARES-SF		0.02	OR = 1.8		Multicenter prospective cohort study	2B
	Freitas-Silva 2010 [77]	70		Goniometer (shoulder internal rotation)	Breast-conserving therapy vs modified radical mastectomy and immediate reconstruction	0.03	OR = 7.23	1.28–17.1	Cross-sectional study	4
	Basu 2020 [60]	342	37 months	Questionnaire was inspired by the Australian BC Family Study		0.04	HR = 1.8	1.1–3.4	Retrospective study	4
	Ahmed 2011 [71]	1,287		Self-report			OR = 1.24	0.95–1.59	Cross-sectional study	4
Adhesive capsulitis	Wong 2021 [75]	135		Restricted passive ROM in 2 or more planes of movement (Goniometer), with normal radiographic findings	Mastectomy with reconstruction	0.021	OR = 3.93	1.23–12.63	Cross-sectional observational study	4
Functional disabilities	Hayes 2010 [39]	287	18 months	QuickDASH and the Functional Assessment of Cancer Therapy Breast questionnaire		0.006	OR = 1.95	0.98–5.21	Prospective study	2B

Abbreviations: *N*: number; CI: confidence interval; OR: odds ratio ; HR: hazard ratio; RR: relative risk; ALND: axillary lymph node dissection; ROM: range of motion; NPRS: numeric pain rating scale.

**Table 6:** Axillary lymph node dissection (ALND) as a risk factor for arm morbidity following breast cancer treatments

Disability	Author	*N*	Follow-up	Measure	Comparison/Comments	*P*-value	OR/HR	CI (95%)	Type of evidence	Evidence level
Lymphedema	Ashikaga 2010 [37]	3,963	36 months	Water displacement		<0.001	OR = 1.90		RCT	1B
	Del Bianco 2007 [82]	341	24 months		ALND vs. SLNB	0.01	OR = 0.48	0.25–1.06	RCT	1B
	Kilbreat 2016 [53]	450	18 months	Circumference and Bioimpedance spectroscopy	>13 lymph nodes	0.07	OR = 2.1	0.9–4.9	Prospective cohort study	2B
	Kwan 2010 [49]	997	20.9 months	Examination by a specialist	No. of dissected lymph nodes		HR = 1.04	1.02–1.07	Prospective cohort study	2B
	Dominick 2013 [61]	2,431	6 years	Norman and colleagues’ validated telephone lymphedema questionnaire		<0.01	OR = 2.08	1.66–2.60	Prospective cohort study	2B
	Park 2008 [63]	450	24 months	Tape measure		0.008	OR = 6.61	1.64–26.57	Prospective study	2B
	Kuijer 2021 [17]	1,037	1 year	CARES-SF		<0.01	OR = 3.6		Multicenter prospective cohort study	2B
	Smoot 2010 [104]	144		Circumference and bioimpedance		<0.005		−7.18–-2.43	Prospective longitudinal study	2B
	Sagen 2014 [83]	391	2.5 years	Water Displacement	ALND vs. SLNB	0.05			Prospective study	2B
	Blanchard 2003 [86]	1,253	12 months	Self-reported	ALND vs. SLNB	<0.001			Prospective study	2B
	Belmonte 2018 [87]	112	5 years	Circumference	ALND vs. SLNB	<0.001		45.8–257.3	Prospective study	2B
	Langer 2007 [81]	635	60 months	Circumference		<0.0001	OR = 0.15	0.08–0.28	Prospective study	2B
	Zou 2018 [47]	387	24 months	Circumference and Norman questionnaire	16><16 lymph nodes	0.09	HR = 5.2	1.6–17.3	Prospective study	2B
	Yen 2009 [89]	1,338	4 years	Self-reported	More than 5 nodes	<0.001	OR = 2.11	4.68–5.61	Population cohort study	2B
	Norman 2009 [105]	631	5 years	Self-reported			HR = 2.61	1.77–3.84	Prospective study	2B
	Chan 2009 [41]		5 years	Arm circumference		0.003	OR = 2.97	1.46–6.03	Matched case-control study	3B
	Herremans 2021 [43]	132	5 years	Arm circumference and bioimpedance spectroscopy	LYMPHA technique vs. ALND	0.036	OR = 0.39	0.16–0.94	Retrospective cross-sectional study	4
	Owusu 2019 [78]	313	-	-		0.008			A descriptive retrospective survey	4
	Kim 2013 [79]	772	8.3 years	Circumference measurements		<0.001	HR = 2.81		Retrospective study	4
	Mccredie 2001 [56]	809		Self-report			OR = 2.4	1.0–5.6	Population-based case-control study	4
	Rebegea 2015 [80]	305			>25 nodes	<0.001	OR = 4.88	2.25–10.58	Cross-sectional study	4
					16–25 nodes	<0.001	OR = 1.85	1.27–2.75		
	Da Costa Vieira 2016 [3]	622	10 years	Self-report	For ≥ 15 lymph nodes	0.017	HR = 9.12	1.15–72.12	Retrospective study	4
	Ahmed 2011 [71]	1,287		Self-report			OR = 3.52	1.32–9.34	Cross-sectional study	4
	Gärtner 2010 [36]	3,104		Self-report		<0.0001	OR = 2.89	2.42–3.45	Cross-sectional study	4
	Crosby 2012 [58]	1,117		From medical records		<0.001	OR = 6.69	2.59–17.29	Retrospective study	4
	Meeske 2009 [35]	494	50 months	Self-report of lymphedema	10 or more lymph nodes		OR = 2.16	1.12–4.17	A case-control study	4
	Langer 2007 [81]	635	60 months	VAS		<0.0001	OR = 0.33	0.20–0.54	Prospective study	2B
Persistent pain	Kramer 2019 [73]	349		SPADI questionnaire			OR = 0.48	0.23–0.98	Cross-sectional study	4
	Mejdahl 2013 [27]	2,411		Developed questionnaire		<0.001	OR = 2.04	1.60–2.61	Cross-sectional study	4
	Chiang 2019 [74]	201		NPRS and Brief Pain Inventory		0.03	OR = 4.33	1.19–15.73	Retrospective cross-sectional study	4
Functional disabilities	Del Bianco 2007 [82]	341	24 months	-	ALND vs. SLNB at 6 months	0.005	OR = 0.47	0.27–0.79	RCT	1B
	Habib 2020 [92]	44	6 weeks	QuickDASH disability score		0.010			Prospective cohort	2B
	Hayes 2010 [39]	287	18 months	QuickDASH and Functional Assessment of Cancer Therapy Breast questionnaire		0.02	OR = 4.81	1.64–14.14	Prospective study	2B
	Kramer 2019 [73]	349		SPADI questionnaire			OR = 0.99	0.54–1.81	Cross-sectional study	4
Decreased range of motion	Ashikaga 2010 [37]	3,963	36 months			<0.001	OR = 1.54		RCT	1B
	Nesvold 2008 [48]	263	47 months	British Columbia Cancer Agency in Canada		0.01	OR = 0.92	0.85–0.98	Prospective study	2B
	Langer 2007 [81]	635	60 months	ROM measurements		0.0002	OR = 0.22	0.15–0.56	Prospective study	2B
	Hack 2010 [59]	316	12 months	Volume measurements		0.003	OR = 3.62	8.48–1.54	Cross-sectional study	4
	Ahmed 2011 [71]	1,287		Self-report			OR = 2.38	1.41–4.03	Cross-sectional study	4

Abbreviations: *N*: number; CI: confidence interval; OR: odds ratio; HR: hazard ratio; ALND: axillary lymph node dissection; SLNB: sentinel lymph node biopsy; VAS: visual analog scale; ROM: range of motion; RCT: randomize control trail.

**Table 7:** Radiotherapy as a risk factor for arm morbidity following breast cancer treatments

Disability	Author	*N*	Follow-up	Measure	Comparison/Comments	*P*-value	OR/RR/HR	CI (95%)	Type of evidence	Evidence level
Persistent pain	Zararli 2009 [32]	299		VAS		0.006	RR = 2.80	1.341–3.821	Prospective study	2B
	Habib 2020 [92]	124	12 months	NPRS		0.03	OR = 2.52	1.13–5.82	Prospective observational study	2B
	Hamood 2018 [24]	410	7.4 years	NPRS	With or without chronic pain	0.003	OR = 2.96	1.43–6.12	A cross-sectional study of a random sample	4
Decreased range of motion	Ashikaga 2010 [37]	3,963	36 months			0.037	OR = 2.48		RCT	1B
	Adriaenssens 2012 [85]	118	3 months	ROM	Abduction after radiation to regional nodes	0.0011			RCT	1B
	Zararli 2009 [32]	299		Physical examination		0.006	RR = 2.46	1.290–4.466	Prospective study	2B
	Kuijer 2021 [17]	1,037	1 year	CARES-SF		0.01	OR = 2.4		Multicenter prospective cohort study	2B
	Kikuuchi 2020 [68]	223	3 months	ROM		0.006	OR = 0.34	0.16–0.73	Prospective study	2B
	Basu 2020 [60]	342	37 months	Questionnaire was inspired by the Australian Breast Cancer Family Study		0.01	HR = 1.1	1.5–2.6	Retrospective study	4
	Ahmed 2011 [71]	1,287		Self-report			OR = 1.72	1.15–2.57	Cross-sectional study	4
Lymphedema	Ashikaga 2010 [37]	3,963	36 months	Water displacement		0.007	OR = 3.47		RCT	1B
	Zou 2018 [47]	387	24 months	Circumference and Norman questionnaire		<0.001	HR = 3.9	2.0–7.5	Prospective study	2B
	Ozcinar 2012 [98]	218	24–82 months	Circumference measurements		<0.001			Prospective observational cohort	2B
	Kilbreat 2016 [53]	450	18 months	Circumferences and Bioimpedance spectroscopy		0.01	OR = 3.7	1.6–8.7	Prospective cohort study	2B
	Ugur 2013 [62]	455	53 months	Tape measure		0.007	OR = 1.83	1.17–2.84	Prospective study	2B
	Bevilacqua 2012 [42]	1,051	5 years	Circumferences	> 6 months	0.0001			Prospective cohort study	2B
	Park 2008 [63]	450	24 months	Tape measure	Axillary radiotherapy	<0.001	OR = 6.73	2.58–17.54	Prospective study	2B
	Kuijer 2021 [17]	1,037	1-year	CARES-SF		0.05	OR = 1.8		Multicenter prospective cohort study	2B
	Dominick 2013 [61]	2,431	6 years	Norman and colleagues’ validated telephone lymphedema questionnaire	Lumpectomy and radiation	<0.01	OR = 1.12	0.91–1.37	Prospective cohort study	2B
					Mastectomy and radiation		OR = 2.02	1.52–2.69		
	Ahmed 2011 [71]	1,287		Self-report			OR = 1.77	0.92–3.40	Cross-sectional study	4
	Hack 2010 [59]	316	12 months	Volume measurements		0.007			Cross-sectional study	4
	Herremans 2021 [43]	132	5 years	Circumferences and bioimpedance spectroscopy	LYMPHA technique VS ALND	0.032	OR = 0.38	0.15–0.91	Retrospective cross-sectional study	4
	Kwan 2002 [84]	744		Self-report		<0.001	OR = 3.1		Cross-sectional	4
	Sueangamiam 2021 [88]	308		Circumferences		0.046	HR = 1.81	1.09–13.28	Cross-sectional study	4
	Da Costa Vieira 2016 [3]	622	10 years	Self-report	Supra-clavicular radiation	0.043	HR = 1.87	1.02–3.45	Retrospective study	4
	Gärtner 2010 [36]	3104		Self-report		0.0005	OR = 1.72	1.30–2.27	Repeated cross-sectional study	4
	Crosby 2012 [58]	1117		From medical record	Immediate breast reconstruction and BMI <25/>25	0.023	OR = 2.23	1.12–4.89	Retrospective study	4

Abbreviations: *N*: number; CI: confidence interval; OR: odds ratio; HR: hazard ratio; RR: relative risk; BMI: body mass index; ALND: axillary lymph node dissection; VAS: visual analog scale; ROM: range of motion; NPRS: numeric pain rating scale; RCT: randomize control trail.

**Table 8:** Chemotherapy as a risk factor for arm morbidity following breast cancer treatments

Disability	Author	*N*	Follow-up	Measure	Comparison/Comments	*P*-value	OR/HR	CI (95%)	Type of evidence	Evidence level
Persistent pain	Kramer 2019 [73]	349		SPADI questionnaire			OR = 0.39	0.18–0.83	Cross-sectional study	4
	Habib 2020 [92]					0.02	OR = 3.39	1.24–10.41	Prospective observational study	2B
Functional disabilities	Kramer 2019 [73]	349		SPADI questionnaire			OR = 0.37	0.18–0.77	Cross-sectional study	4
	Khan 2012 [33]	85		FIM Measurement		< 0.001			Prospective study	2B
Decreased range of motion	Woo 2018 [45]	430		Having restricted ROM ≥ 30° in compared to unaffected side	Different modalities of breast reconstructions	0.002	OR = 5.578	1.83–16.95	Prospective study	2B
	Ashikaga 2010 [37]	3,963	36-months			0.003	OR = 0.73		RCT	1B
Lymphedema	Kilbreat 2016 [53]	450	18 months	Circumferences and bioimpedance spectroscopy (BIS)	Taxane-based chemotherapy	0.04	OR = 2.3	1.0–5.2	Prospective cohort study	2B
					Chemotherapy	0.08	OR = 2.2	0.9–5.3		
	Rupp 2019 [99]	385		LBCQ-D and SDBC-D	Adjuvant chemotherapy	0.005	OR = 2.5	0.21–0.76	Retrospective study	4
	Ahmed 2011 [71]	1,287		Self-report			OR = 3.05	1.75–5.30	Cross-sectional study	4
	Bevilacqua 2012 [42]	1,051	5 years	Circumferences	At 0–6 months	0.0001			Prospective cohort	2B
	Herremans 2021 [43]	132	5 years	Circumferences and bioimpedance spectroscopy	LYMPHA technique VS ALND	0.030	OR = 0.38	0.15–0.90	Retrospective cross study	4
	Da Costa Vieira 2016 [3]	622	10 years	Self-report		0.051	HR = 0.5	0.21–1.17	Retrospective study	4
	Das 2015 [55]	199		Self-reported	Tamoxifen therapy only	0.02	OR = 2.31	1.13–4.71	Cross-sectional	4
	Gärtner 2010 [36]	3104		Self-report		<0.0001	OR = 1.92	1.66–2.22	Repeated cross-sectional study	4
	Norman 2009 [105]	631	5 years	Self-reported			HR = 1.46	1.02–2.04	Prospective study	2B

Abbreviations: *N*: number; CI: confidence interval; OR: odds ratio; HR: hazard ratio; BMI: body mass index; ALND: axillary lymph node dissection; VAS: visual analog scale; ROM: range of motion; NPRS: numeric pain rating scale; FIM: functional independence measure; RCT: randomize control trail.

## Risk factors for the morbidity of the arm divided into three categories: personal factors, factors related to the type of surgery, and factors related to the type of oncological treatment

### Personal risk factors for arm morbidities

#### Age

Young age was evaluated in seven studies, including 18,272 patients. Young age was found to be associated with persistent pain or more severe pain [23–29], radiation-associated pain [30, 31], paresthesia, loss of strength [32], poor functioning [33, 34] and lymphedema [35, 36]. Younger women (≤49) were found to have larger tumor sizes and were associated with having a mastectomy in contrast to a lumpectomy [37]. Furthermore, young age was found to be a risk factor for axillar web syndrome (AWS), a complication that often develops within weeks following BC surgery and may present as one or more visible or palpable tight cords of tissue in the axilla [38].

Age over 50 was evaluated in seven trials comprising 7,914 patients, and was found to be associated with functional decline [39], ROM deficit [40], lymphedema [37, 41–43], and pain six months after surgery [44]. Older women who underwent different modalities of breast reconstruction were at higher risk of limitation in ROM [45] (See Tables 2, 3).

#### Body mass index (BMI)

BMI was evaluated in 15 trials comprising 9,025 patients. BMI above 30 was associated with higher risk for lymphedema [17, 35, 41, 42, 46–58], functional disability [59], and decreased ROM [17]. BMI above 25 was negatively and significantly related to shoulder ROM deficit, function decline [40, 60], lymphedema [48, 61–64], and pain [65] (See Table 4).

#### BC history

BC history was evaluated in one trial that included 489 participants. A high number of previous operations was found to be a risk factor for persistent pain six months after surgery [44].

#### Preoperative ROM deficit

Preoperative ROM deficit was evaluated in two trials including 192 participants. A history of shoulder problems and restricted humeral extension preoperatively was found to be associated with persistent pain [66, 67].

#### Preoperative pain

Preoperative pain was evaluated in three trials including 20,459 participants. Preoperative pain in the breast area was found to associated with persistent pain and functional disability [25, 67], in addition to chronic pain conditions [44].

#### Functional decline before surgery

Functional decline prior to surgery may cause more functional disabilities after surgery, as well as a decrease in ROM [67].

#### Emotional support

Lack of emotional support was evaluated in one trial that included 316 patients. Lack of a partner was found to be associated with persistent postoperative pain six months after surgery [59].

#### Low educational attainment

Low educational status was found to be associated with ROM restrictions [65], and lymphedema [50, 56]. On the other hand, a different study found associations between a high level of education and lymphedema [49].

#### Low socioeconomic status

Defined by income was found to have an effect on upper body functional impairments [39]. In addition, patients who reported financial distress were found to be at a higher risk of lymphedema one year after surgery [17].

#### Follow-up by public health services

After cancer treatment, receiving care from public health services was associated with a higher degree of functional disability compared to patients treated in private clinics [34].

#### Treatment on the dominant side

It was found that treatment to the contralateral side can have an effect on upper body functional impairments [39] and ROM of the effected arm [68].

#### Breast cancer stage at diagnosis

Regionally advanced cancer was associated with chronic pain [24]. Cancer Stage III/IV was found to be a predictor of neuropathic pain [69], while more advanced disease was found to cause arm morbidity [70], including pain, limited ROM [59], and lymphedema [17, 50, 62, 63, 71]. Aggressive tumors were found to be associated with poor levels of functioning [33].

#### Previous smoking

Smoking was found to be a risk factor for persistent pain six months after surgery [59].

#### Diabetes mellitus

Diabetes was found to be a prognostic factor for arm and shoulder morbidity, including stiffness, edema, numbness, and pain [60].

#### A history of hypertension

Hypertension was found to be a risk factor for lymphedema [35].

#### Anxiety

Anxiety was found to be a predictor of neuropathic pain [69]. Additionally, arm symptoms, lower emotional functioning and insomnia presented one month following surgery were identified as significant risk factors for persistent pain [72].

#### African American ethnicity

African American ethnicity was associated with an increased risk of lymphedema [49] and higher pain levels [73], and radiation-associated pain [30]. Non-European ethnicity was identified as risk factors for moderate to severe persistent pain after surgery [74].

#### Comorbid diseases

Comorbidity were significantly correlated with the development of lymphedema [50, 71] and pain [30].

#### Time after surgery

The time elapsed since surgery had a significant correlation with the development of lymphedema [50].

### Surgery-related risk factors for arm morbidities

#### The effect of a lumpectomy vs. a mastectomy surgery

The effect of the type of surgery was evaluated in 16 articles that comprised 7,392 patients. Chronic pain was found to be positively associated with a mastectomy compared to breast-conserving surgery [24, 48]. A mastectomy without reconstruction was found to be related to flexion and abduction ROM loss at 12 months after surgery [40] and functional impairments [39]. Breast-conserving surgery was associated with long-term paresthesia [32]. In addition, both radical mastectomy and modified radical mastectomy were found to be risk factors for lymphedema [47, 59, 64], with both causing restricted shoulder ROM [17, 48, 60]. A mastectomy was also found to be a risk factors for adhesive capsulitis [75]. A lumpectomy was found to be related to a decline in the level of activities of daily living [76]. Contrary to expectations, it was found that breast-conserving therapy caused a higher prevalence of restricted internal rotation compared to modified radical mastectomy and immediate breast reconstruction [77] (see Table 5 and Figure 4A).

#### Axillary lymph node dissection (ALND)

ALND was found to cause higher lymphedema frequency [3, 17, 42, 43, 47, 49, 56, 63, 78–82], persistent pain [27, 73, 74, 81], reduced muscle strength and restricted ROM compared to SLNB [1, 25, 39, 46, 59, 65, 66, 70, 81–87], in addition to seroma formation and infections [86]. A dissection of more than 20 lymph nodes increases the risk of lymphedema [58, 61, 88], and even 6–10 dissected nodes may cause lymphedema, pain, and ROM restrictions [35, 67, 71, 89]. In addition to breast-conserving surgery or a mastectomy, ALND was found to be a predictor for neuropathic pain [69]. Furthermore, ALND was associated with more numbness and tingling compared to SLNB [37], and a higher risk for AWS [38] (See Table 6 and Figure 4B).

#### Breast reconstruction surgery

Breast reconstruction surgery was evaluated in three trials comprising 766 patients. Regardless of the specific reconstructive modality, pain and functional deficits following surgery are extremally common [45, 90], and were identified as risk factors for moderate to severe persistent pain after surgery [74]. Reconstruction surgery was found to be a risk factors for adhesive capsulitis [75], but not a risk factor for lymphedema [58, 91].

#### Pain during hospitalization

Pain during hospitalization was evaluated in four trials comprising 597 patients. Greater acute postoperative pain was found to be an indicator of persistent pain [25, 92]. Higher pain levels in the early postoperative period were found to correlate with restricted shoulder ROM [93].

#### Postoperative infection

Postoperative infection was found to cause pain and functional disability [59]. Trauma or infection in the affected ipsilateral arm after surgery were found to be risk factors associated with lymphedema [41, 42, 50, 52, 54, 62], and cellulite events [46].

#### Axillary web syndrome (AWS)

AWS was identified as a risk factor for reduced function, ROM impairments, pain, and lymphedema at 18 months [94].

#### The number of positive axillar lymph nodes

The number of dissected lymph nodes was related to loss of flexion and abduction ROM [40] and lymphedema [47, 48, 50, 70, 89]. Furthermore, the number of positive nodes were found to be a cause of reduced ROM in reconstruction surgeries [45].

#### Lymphedema

Lymphedema was found in a meta-analysis as the most significant risk factor for chronic pain [65], diminished quality of life and functional impairments [39, 95]. In addition, early edema in the first six months postoperatively was found to be a risk factor for lymphedema [42].

Limitation in shoulder movement due to pain, and lymphedema was found to cause functional limitations [47].

Postoperative seroma was found to be a risk factor for lymphedema [42].

Capsule invasion of the tumor was found to be a risk factor for lymphedema [96].

Damage to the intercostobrachial nerve was found to be a predictor of neuropathic pain [69].

Hospital skin puncture (defined as any intentional puncture by a health professional or patient of the patient’s skin in the hand or arm on the ipsilateral side with a needle for any purpose, including finger prick glucose testing) was found to be a risk factor for lymphedema [64].

### Treatment-related risk factors for arm morbidities

#### Radiation therapy

The effect of radiation therapy was evaluated in 25 trials including 24,195 patients and was found to be a risk factor for persistent pain [24, 32, 65, 92, 97], shoulder stiffness [32, 60, 71], decreased ROM [17, 37, 68], and lymphedema [3, 17, 36, 37, 42, 43, 46, 47, 61–63, 70, 84, 88, 98] (See Table 7 and Figure 5A).

#### Chemotherapy

Chemotherapy was evaluated in 13 trials including 12,406 patients. Neoadjuvant and adjuvant chemotherapy were found to be risk factors for chronic pain [65, 73, 92], functional disabilities [33, 73], and lymphedema [43, 53]. Neoadjuvant chemotherapy was found to be a risk factor for limitations in ROM in patients that had undergone different modalities of breast reconstructions [45]. Adjuvant chemotherapy was found to be a risk factor for the appearance of lymphedema [3, 42, 71, 99], especially tamoxifen [55], although taxane-based chemotherapy were not found to increase the risk of lymphedema compared to patients receiving no chemotherapy or non-taxane adjuvant chemotherapy. Those treated with docetaxel may experience mild swelling, but this does not translate into subsequent lymphedema [100] (see Table 8 and Figure 5B).

Hormonal therapy was found to be a risk factor for persistent pain [76].

A description of the number of studies found in the literature review for each risk factor, in relation to the different morbidity: lymphedema, decreased range of motion, decreased function and prolonged pain, is shown in Supplementary Figure 1.

## DISCUSSION

Breast cancer surgery and treatments are known to cause long-term physical morbidity of the arm. These morbidities include prolonged pain or chronic pain [23, 29], limitations in ROM, decreased function [1, 2, 6, 59, 84] and lymphedema [8, 16, 101]. All these morbidities can last for months or even years after surgery [1, 2], and cause an additional burden on the primary cancer treatment.

Our systematic review aimed to comprehensively analyse the risk factors for these four long-term arm morbidities that are common after BC surgeries and treatments. The analysis encompassed various elements, such as surgical and oncological factors, patient medical conditions, lifestyle habits, work-related factors, family support, and emotional well-being.

This review revealed 29 primary risk factors associated with arm morbidity after BC treatment. These factors ranged from surgical procedures such as mastectomy and ALND to medical considerations like chemotherapy, radiation therapy, and cancer stage. Additionally, patient-related factors such as BMI, smoking, anxiety levels, and emotional support were found to influence the development of morbidity.

Age emerged as a significant factor impacting morbidity. Older age was found to be a risk factor for lymphedema, functional limitation and ROM deficits, while young age was found to be associated with prolonged pain, as found in a meta-analysis by Wang et al. [25]. Contrary to the report of Sipila et al., who found that older age (over 70) is associated with prolonged pain [44].

Additional risk factors contributing to prolonged arm morbidity following BC have been investigated by various researchers, each focusing on specific morbidities. Leysen et al. and Wang et al., in their systematic review and meta-analysis, identified several risk factors associated with prolonged pain, including BMI>30, lower education levels (<12–13 years), younger age, lymphedema, smoking, axillary lymph node dissection (ALND), chemotherapy, hormonal therapy, radiation, greater acute postoperative pain, and preoperative pain [25, 65].

Similarly, DiSipio et al., in their literature review and meta-analysis, reported risk factors for lymphedema, which include ALND or a greater number of lymph nodes dissected, mastectomy surgery, and being overweight or obese [101]. Torgbenu et al. also emphasized BMI>25 as a significant risk factor for lymphedema [8].

In this comprehensive review, additional risk factors were found to be associated with prolonged arm morbidity following BC treatments. These factors encompass various aspects, such as low socioeconomic level, African-American ethnicity, lack of monitoring by public health services, insufficient emotional support, anxiety, comorbidity, advanced cancer stage, preoperative functional decline, preoperative ROM deficit, postoperative infection, trauma to the affected arm, AWS, the number of positive lymph nodes, and postoperative seroma.

The identification of these diverse risk factors enhances our understanding of the multifactorial nature of arm morbidity in breast cancer patients. By acknowledging and recognizing these risk factors, healthcare providers can develop more targeted interventions and personalized treatment plans. This, in turn, leads to improved outcomes and a reduction in the burden of prolonged arm morbidity for patients. Such invaluable insights are instrumental in optimizing rehabilitation strategies and enhancing patient care, ultimately resulting in an improved quality of life for breast cancer survivors.

Our findings underscore the importance of utilizing risk factor information to create predictive tools and tailored treatment plans. By addressing these risk factors proactively, we have the potential to reduce future morbidity, improve patient outcomes, and significantly enhance the overall recovery process for breast cancer patients.

## MATERIALS AND METHODS

This systematic review was reported in accordance with the Preferred Reporting Items for Systematic Reviews and meta-analysis guidelines (PRISMA) [102].

To identify relevant studies regarding risk factors for persistent pain, lymphedema, decreased range of motion (ROM) and function in cancer survivors. A systematic search of the literature was conducted in the databases PubMed, Cochrane Central Register of Controlled Trials, MEDLINE, and Pedro up to February 2022, using MeSH terms and free key words. Authors were contacted if the full texts of their studies could not be retrieved.

Search terms associated with BC-related arm morbidity were used (e.g., risk factors, arm morbidity, chronic/persistent pain, function decline, function disability, lymphedema, lymphedema, ROM deficit, decreased/diminished ROM). We searched for different risk factors, namely, BMI, age, mastectomy, axillary dissection, breast reconstructions, chemotherapy, radiotherapy, BC history, pre-operative pain, pre-operative functional decline, pre-operative ROM deficit, pain during hospitalization, comorbidity, race, socioeconomic status, education, postoperative complications such as infection or serum, emotional support, and anxiety levels.

### Inclusion criteria

The inclusion criteria were as follows: (1) Studies and systematic reviews and meta-analyses published between the years 2000–2021; (2) full text in English; (3) studies that examined risk factors for arm morbidity in patients diagnosed with breast cancer (BC); (4) studies on patients who had undergone surgical and oncological treatments; and 5) articles in which the data for determining risk factors were available.

### Exclusion criteria

The exclusion criteria were as follows: (1) Case reports and study protocols; (2) patients with a diagnosis of cancer recurrence; (3) subjects diagnosed with cancers other than BC; (4) articles in which persistent pain, lymphedema, decreased range of motion, ROM and function were not presented as an outcome; (5) studies with less than 30 participants; and (6) systematic reviews and meta-analyses.

Two independent reviewers (IK, DS) screened the titles and abstracts of studies identified as eligible. Articles considered potentially relevant were obtained, and the full articles were screened for inclusion by the same two independent reviewers. IK and DS resolved disagreements by consensus. The tables reported in the review represent studies in which the researchers found positive relationships between the various risk factors for long-term morbidity of the arm (including: pain, decreased function, limitation of range of motion or lymphedema).

### Quality assessment

The level of evidence was evaluated according to the Levels of Evidence for Therapeutic Studies [103]. The risk of bias was evaluated using the National Institutes of Health (NIH) quality assessment tool for each type of study: for RCTs, using the Quality Assessment of Controlled Intervention Studies, for non-RCTs, using the Quality Assessment Tool for Observational Cohort and Cross-Sectional Studies, both of which include 14 items.

## CONCLUSIONS

In conclusion, gaining a comprehensive understanding of the risk factors associated with arm morbidity following breast cancer treatment offers invaluable insights to healthcare professionals, enabling them to optimize patient care and rehabilitation strategies effectively. By identifying and proactively addressing these risk factors, we can strive to minimize the impact of long-term physical morbidity and enhance the overall quality of life for breast cancer survivors. Through this proactive approach, we can foster better outcomes and support the well-being of those who have overcome breast cancer challenges.

The main risk factors for lymphedema include the number of removed lymph nodes, BMI, mastectomy, disease stage, radiation, chemotherapy, infection, and post-surgery trauma. Decreased ROM is linked to radiation, mastectomy, ALND, and BMI >30. Decreased function is influenced by ALND, age <50, BMI >25, and chemotherapy. Prolonged pain is associated with age <50, radiation, chemotherapy, ALND, disease stage, mastectomy, and intense preoperative pain. Future research should use these risk factors to develop predictive tools and tailored treatment protocols for improved patient outcomes.

## SUPPLEMENTARY MATERIALS



## Abbreviations

BC: Breast cancer
BMI: body mass index
PT: physical therapy
ALND: axillary lymph node dissection
SLNB: sentinel lymph node biopsy
VAS: visual analog scale
ROM: range of motion
NPRS: numeric pain rating scale
AWS: axillary web syndrome
CI: confidence interval
OR: odds ratio
HR: hazard ratio
RR: relative risk
RCT: randomize control trail
N: number
