# Supplementary material for: Risk factors for long-term arm morbidities following breast cancer treatments: A systematic review
Source: Oncotarget. 2023 Dec 1;14:921–42. doi: 10.18632/oncotarget.28539 (PMC10691815; doi:10.18632/oncotarget.28539)
Supplement: Supplementary file 1 [file oncotarget-14-28539-s001.pdf]

Risk factors for long-term arm morbidities following breast cancer treatments: A systematic review

SUPPLEMENTARY MATERIALS

	Risk factor	Lymphedema	Decreased ROM	Decreased function	Prolonged / chronic pain
Personal factors	Age <55	2		3	10
	Age >55	4	2	1	2
	BMI>25	22	3	2	2
	Socio-economic level	1		1	
	Low educational attainment	3			
	Cancer stage	5	1	1	3
	Comorbid diseases	2			1
	Anxiety				1
	Smoking				1
	African American	1			2
	Recurrence of BC				1
	Preoperative pain			1	2
	Preoperative functional decline		1	1	
	Preoperative ROM deficit		1		2
	Lack of emotional support				1
Surgery related factors	Mastectomy	6	6	1	3
	ALND	26	4	4	4
	Breast reconstruction		1	1	1
	Extreme pain during hospitalization		1		3
	Treatment on the dominant side		1	1	
	Not monitored by public services			1	
	Postoperative infection	6		1	1
	Postoperative trauma	6			
	Axillar web syndrome	1	1		1
	The number of positive nodes	5	1		
	Postoperative seroma	1			
Treatment related factors	Chemotherapy	9	2	2	2
	Radiotherapy	17	7		4
	Hormonal therapy				1

Supplementary Figure 1: A summary of all the significant risk factors for arm morbidity found in the literature, reported by the number of studies found. Describes the number of studies found in the literature review for each risk factor, in relation to the different morbidity: lymphedema, decreased range of motion, decreased function, and prolonged pain. Legend: Red: one study, yellow: 2–4 studies, light green: 5–15 studies, green: 16–19 studies, dark green: over 20 articles. Abbreviation: CI: confidence interval.
